# The duration of estrogen treatment before progesterone application does not affect neonatal and perinatal outcomes in frozen embryo transfer cycles

**DOI:** 10.3389/fendo.2023.988398

**Published:** 2023-07-21

**Authors:** Junwei Zhang, Mingze Du, Zhongkai Wang, Sheling Wu, Yichun Guan, Lijun Sun

**Affiliations:** ^1^ The Reproductive Center, The Third Affiliated Hospital of Zhengzhou University, Zhengzhou, Henan, China; ^2^ Obstetrics and Gynecology, The Third Affiliated Hospital of Zhengzhou University, Zhengzhou, Henan, China

**Keywords:** frozen embryo transfer, artificial cycle, duration of estrogen, gestational diabetes mellitus, neonatal outcomes

## Abstract

**Objective:**

To explore whether the duration of estrogen treatment before progesterone application affects neonatal and perinatal outcomes in artificial frozen embryo transfer (FET) cycles.

**Methods:**

This was a retrospective cohort study. Patients who underwent FET via artificial cycles and delivered a singleton live birth between January 2015 and August 2019 were included in the analysis. According to the duration of estrogen treatment before progesterone application, we divided the cycles into four groups: ①≤12 days, ②13-15 days, ③16-19 days, and ④≥20 days. The ‘≤12 days group’ was considered the reference group. The main outcome measures were preterm birth (PTB), small-for-gestational age (SGA), low birth weight (LBW), macrosomia, large-for-gestational age (LGA), gestational diabetes mellitus (GDM), gestational hypertension, premature rupture and placenta previa.

**Results:**

Overall, 2010 FET cycles with singleton live births were included for analysis. Cycles were allocated to four groups according to the duration of estrogen treatment before progesterone application: ①≤12 days (n=372), ②13-15 days (n=745), ③16-19 days (n=654), ④≥20 days (n=239). The neonatal outcomes, including PTB, SGA, LBW, macrosomia and LGA, were comparable among the groups (P=0.328, P=0.390, P=0.551, P=0.565, P=0.358). The rates of gestational hypertension, premature rupture and placenta previa (P=0.676, P=0.662, P=0.211) were similar among the groups. The rates of GDM among the four groups were 4.0% (15/372), 6.7% (50/745), 6.4% (42/654), and 11.3% (27/239), with statistical significance (P=0.006). After multiple logistic regression analysis, the duration of estrogen treatment did not affect the rate of GDM or other outcomes.

**Conclusion:**

The estrogen treatment duration before progesterone application does not affect neonatal and perinatal outcomes in single frozen blastocyst transfer cycles.

## Introduction

In the past decade, the number of frozen embryo transfer (FET) cycles has substantially increased. Currently, up to half of embryos are cryopreserved, and FET is performed (1). This trend may be related to the improvement and development of vitrification technology and a rapid rise in single embryo transfer (ET), combined with the development of preimplantation genetic tests (PGTs) (2–4). In addition, current studies have shown that FET can reduce *in vitro* fertilization (IVF) complications, such as ovarian hyperstimulation syndrome, increase the cumulative live birth rate, and improve perinatal and offspring outcomes (5–7). How to improve the live birth rate of FET cycles and ensure the safety of mothers and offspring is the focus of our research. Ensuring the synchronization of embryo and endometrial development is a key step in obtaining pregnancy. However, the optimal endometrial preparation protocols for FET are still a topic of constant debate (8–10). The artificial cycle is mainly suitable for patients with abnormal ovulation or nonovulation. The application of exogenous estrogen and progesterone can promote the development of the endometrium and promote the synchronization of the development of the endometrium and the embryo. Preparing the endometrium in artificial cycles, which can flexibly arrange the transplantation time and reduce the number of monitoring procedures, is widely used in clinical practice (1). Therefore, how to optimize this protocol, improve the clinical outcome and ensure the safety of the perinatal period is the focus of our attention. At present, there are studies exploring the optimal duration of estrogen treatment before progesterone application; however, most studies focus on the pregnancy rate or live birth rate (11–15). To the best of our knowledge, only two studies have analyzed the relationship between the duration of estrogen treatment and offspring and perinatal safety (13, 15). However, the conclusions of the two studies are not consistent. Due to the differences in the included population and groupings, it is necessary to further analyze the effect of estrogen use time on neonatal and perinatal outcomes. Therefore, the purpose of this study was to analyze whether the duration of estrogen treatment before progesterone application affects neonatal and perinatal outcomes in artificial FET cycles.

## Materials and methods

### Study design and population

This was a retrospective cohort study conducted in the reproductive center of the Third Affiliated Hospital of Zhengzhou University. Permission to conduct this study was obtained from the Ethics Committee of the Third Affiliated Hospital of Zhengzhou University. The outcomes of patients who underwent the first FET of whole embryo freezing and delivered a singleton live birth between January 2015 and August 2019 were included for potential analysis. All included patients had the endometrium prepared for ET through artificial cycles. All included patients followed the oral glucose tolerance test and insulin release test, and patients with abnormal blood glucose or insulin were excluded. We excluded cycles with oocyte donation(n=92), PGT(n=176) and incomplete data(n=45). In addition, studies have shown that thin endometrium thickness (EMT) may have an impact on offspring weight and perinatal outcomes (16–19). To reduce the influence of thin EMT, cycles with EMT ≤7 mm within 10 days of the initiation of estrogen administration were excluded from the analysis(n=81).

### Endometrial preparation and ET

The controlled ovarian stimulation protocols and IVF/ICSI process have been described in our previous studies (5, 20, 21). In the present study, all patients underwent artificial cycles for FET. Vaginal ultrasound examination was performed on the 2nd/3rd day of the menstrual cycle, 2-3 mg of estradiol valerate was taken orally three times daily (Bayer Co. Germany), and vaginal ultrasound examination was performed 7 days later. The drug dose was adjusted according to the thickness of the endometrium (up to 9 mg per day). The duration of estrogen treatment continued for at least 10 days. If conditions were appropriate (EMT>7 mm, serum estrogen level ≥150 pg/ml and serum progesterone < 1.5 ng/ml) and the patient’s schedule was feasible, the progesterone exposure day was formulated. Routine corpus luteum support, namely, oral dydrogesterone (2 times daily, 10 mg once) (Abbott Co. America) and intravaginal administration of 90 mg of a progesterone sustained-release vaginal gel (Merck Co. Germany), was given. Transfer 1-2 cleavage-stage embryos 3 days after endometrial progesterone conversion or transfer 1 blastocyst 5 days after endometrial transformation. Vitrified frozen embryos were thawed according to our reproductive standard procedure (22). Specifically, embryos were quickly released and immersed in thawing solution for 1 minute at 37°C. Then, embryos were moved into dilution solution for 3 minutes, followed by two steps in washing solution for 3 min each at room temperature. Thawed embryos were incubated for 2 h before transfer. Embryo transplantation was carried out by abdominal ultrasound. Corpus luteum support was performed 55 days after transplantation if pregnancy occurred.

According to the duration of estrogen treatment before progesterone application, we divided the cycles into four groups: ①≤12 days, ②13-15 days, ③16-19 days, and ④≥20 days. The ‘≤12 days group’ was considered the reference group. The grouping was mainly based on the interquartile range of estrogen treatment.

### Outcome measures and definition

Outcome measures of this study included neonatal and perinatal outcomes. Small-for-gestational age (SGA) was defined as a birth weight less than the 10th centile for gestational age (23). Large-for-gestational age [LGA, defined as a birth weight greater than the 90th centile of the sex-specific birth weight (23)]. The weight criteria refer to the weight of Chinese newborns (24). Other neonatal outcomes included preterm birth (PTB) (defined as a birth that takes place after 28 weeks and before 37 completed weeks of gestational age), low birth weight (LBW, defined as a neonatal birth weight less than 2500 g) (23) and macrosomia (defined as a neonatal birth weight more than 4000 g).

We also analyzed the rate of pregnancy-related complications among the following groups: ①Hypertensive disorders of pregnancy, including chronic hypertension, preeclampsia-eclampsia, preeclampsia superimposed on chronic hypertension, and gestational hypertension. ②Gestational diabetes mellitus: GDM was defined as carbohydrate intolerance of variable severity with onset or first recognition during pregnancy as determined from the diagnosis in the obstetrical medical record. ③ Premature rupture of membrane (PROM): PROM was defined as rupture of the amniotic membranes before the onset of labor, including PROM of term and preterm PROM. ④ Placenta previa: Placenta previa was used to describe a placenta that is implanted over or very near the internal cervical os (7).

### Statistical analysis

All the data in this article were obtained from the electronic medical record system of the reproductive center of the Third Affiliated Hospital of Zhengzhou University.

The one-sample Kolmogorov–Smirnov test was used to check for normality of continuous variables. Continuous variables with abnormal distributions are expressed as the median (P25, P75), and the between-group differences were assessed by the Wilcoxon rank sum test. Categorical variables are represented as the number of cases (n) and percentage (%). The means from chi-square analyses were used to assess the differences between groups with Fisher’s exact test when necessary. For the neonatal and perinatal outcomes of this study, multiple logistic regression was used to adjust for the baseline characteristics. Adjusted odds ratios (AORs) with 95% confidence intervals (CIs) were calculated. P<0.05 was considered to be statistically significant. Statistical management and analyses were performed usingSPSS software, version 22.0.

## Results

### Study population

Overall, 2010 FET cycles with singleton live births from January 2015 to August 2019 were included for analysis. Cycles were allocated to four groups according to the duration of estrogen treatment before progesterone application,

①≤12 days (n=372), ②13-15 days (n=745), ③16-19 days (n=654), ④≥20 days (n=239).

### Baseline characteristics

The details of the baseline and cycle characteristics among the four groups are described in **Table 1**. The endometrial thickness was different among groups on the first day of progesterone administration (≤12 days, 9.5 (8.7, 10.5); 13-15 days, 9.0 (8.3, 10.0); 16-19 days, 8.5 (8.0, 9.3); ≥20 days, 8.0 (7.4, 8.7, P<0.001). Apart from this, there were significant differences in maternal age at oocyte retrieval (P=0.017), paternal age (P=0.024), duration of infertility (P=0.002), body mass index (BMI) (P=0.011), gravidity (P=0.023), parity (P=0.019), number of previous miscarriages (P=0.008), infertility diagnosis (P=0.014) and AMH (P<0.001). Other basic characteristics, including type of infertility (P=0.062), PCOS ratio (P<0.001), basal serum FSH (P=0.384), basal antral follicle count (P=0.059), method of ART (P=0.648), number of transferred embryos (P=0.838) and type of transferred embryos (P=0.191), were similar among groups.

**Table 1:** Comparison of demographic characteristics among the four groups.

Item	Duration of estrogen treatment before progesterone administration (days)				*P* value
	≤12	13-15	16-19	≥20	
No. of cases	372	745	654	239	
Maternal age (year)	31.0 (28.0,34.0)	30.0 (28.0,34.0)	30.0 (28.0,33.0)	30.0 (27.0,33.0)	0.017
Paternal age (year)	31.0 (29.0,35.0)	31.0 (28.0,35.0)	31.0 (28.0,35.0)	30.0 (28.0,34.0)	0.024
Body mass index (kg/m2)	24.1 (22.0,26.4)	23.7 (21.5,26.0)	23.6 (21.5,25.6)	23.4 (21.0,25.5)	0.011
Gravidity	0 (0,1)	1 (0,2)	1 (0,2)	1 (0,2)	0.023
Parity	0 (0,0)	0 (0,1)	0 (0,0)	0 (0,0)	0.019
Number of previous miscarriages	0 (0,0)	0 (0,0)	0 (0,0)	0 (0,1)	0.008
Duration of infertility (year)	3.0 (2.0,6.0)	3.0 (1.0,5.0)	3.0 (1.0,4.0)	3.0 (1.0,5.0)	0.002
Type of infertility (%)					0.062
Primary infertility	50.3 (187/372)	45.2 (337/745)	46.8 (306/654)	39.3 (94/239)	
Secondary infertility	49.7 (185/372)	54.8 (408/745)	53.2 (348/654)	60.7 (145/239)	
Infertility diagnosis (%)					0.014
Tubal factor	29.0 (108/372)	33.0 (246/745)	33.0 (216/654)	41.4 (99/239)	
Male factor	16.7 (62/372)	20.8 (155/745)	23.2 (152/654)	17.6 (42/239)	
Male+female factors	21.0 (78/372)	18.0 (134/745)	17.7 (116/654)	15.5 (37/239)	
Others	33.3 (124/372)	28.2 (210/745)	26.0 (170/654)	25.5 (61/239)	
PCOS ratio	15.9 (59/372)	20.0 (149/745)	15.1 (99/654)	32.6 (78/239)	<0.001
Basal FSH (IU/L)	6.5 (5.0,8.2)	6.2 (5.0,7.7)	6.2 (4.8,7.6)	6.4 (5.1,7.7)	0.384
AMH (ng/ml)	3.5 (1.6,6.7)	4.1 (2.1,7.1)	4.6 (2.5,7.5)	5.2 (2.9,7.9)	<0.001
Basal antral follicle count	17.0 (11.0,24.0)	19.0 (12.5,24.0)	20.0 (13.0,24.0)	20.0 (15.0,24.0)	0.059
Method of ART (%)					0.648
IVF	76.9 (286/372)	74.1 (552/745)	73.4 (480/654)	73.6 (176/239)	
ICSI	23.1 (86/372)	25.9 (193/745)	26.6 (174/654)	26.4 (63/239)	
Endometrial thickness on the first day of progesterone administration (mm)	9.5 (8.7,10.5)	9.0 (8.3,10.0)	8.5 (8.0,9.3)	8.0 (7.4,8.7)	<0.001
No. of transferred embryos					0.838
1	52.7 (196/372)	54.1 (403/745)	54.1 (354/654)	56.5 (135/239)	
2	47.3 (176/372)	45.9 (342/745)	45.9 (300/654)	43.5 (104/239)	
Type of transferred embryos					0.191
Cleavage embryo	33.1 (123/372)	33.4 (249/745)	28.9 (189/654)	28.5 (68/239)	
Blastocyst	66.9 (249/372)	66.6 (496/745)	71.1 (465/654)	71.5 (171/239)	

Data are presented as median (P25, P75) for continuous variable and %(n/N) for categorical variable.

### Neonatal and perinatal outcomes

The specific rates of neonatal and perinatal outcomes are described in **Table 2**. The mean neonatal birth weight was comparable among groups (≤12 days, 3500 (3150, 3800); 13-15 days, 3500 (3150, 3800); 16-19 days, 3405 (3100, 3800); ≥20 days, 3490 (3100, 3700), P=0.168). The neonatal sex ratio (P=0.693) and gestational weeks at delivery were similar between the groups (P=0.940). The rates of PTB, LBW, SGA, macrosomia, LGA, gestational hypertension, premature rupture and placenta previa were comparable among the groups (P=0.328, P=0.551, P=0.390, P=0.565, P=0.358, P=0.676, P=0.662, P=0.211). The rate of gestational diabetes mellitus was different among groups, which was 4.0% (15/372), 6.7% (50/745), 6.4% (42/654), and 11.3% (27/239), P=0.006.

**Table 2:** Comparison of neonatal and perinatal outcomes among groups.

	Duration of estrogen treatment before progesterone administration (days)				*P value*
	≤12	13-15	16-19	≥20	
No. of cases	372	745	654	239	
Neonatal outcomes					
Neonatal birth weight(g)	3500 (3150,3800)	3500 (3150,3800)	3405 (3100,3800)	3490 (3150,3700)	0.168
Neonatal sex (%)					0.693
Male	56.2 (209/372)	54.2 (404/745)	53.4 (349/654)	51.5 (123/239)	
Female	43.8 (163/372)	45.8 (341/745)	46.6 (305/654)	48.5 (116/239)	
Gestational weeks at delivery(week)	39 (38,40)	39 (38,40)	39 (38,40)	39 (38,40)	0.940
Preterm birth (%)	6.7 (25/372)	7.5 (56/745)	9.2 (60/654)	10.0 (24/239)	0.328
Low birth weight (%)	5.6 (21/372)	4.6 (34/745)	6.3 (41/654)	5.0 (12/239)	0.551
Small-for-gestational age (%)	4.3 (16/372)	4.4 (33/745)	5.2 (34/654)	2.5 (6/239)	0.390
Macrosomia (%)	13.4 (50/372)	16.0 (119/745)	15.1 (99/654)	13.0 (31/239)	0.565
Large-for-gestational age (%)	28.0 (104/372)	26.6 (198/745)	25.4 (166/654)	21.8 (52/239)	0.358
Pregnancy-related complications					
Gestational diabetes mellitus (%)	4.0 (15/372)	6.7 (50/745)	6.4 (42/654)	11.3 (27/239)	0.006
Gestational hypertension (%)	7.3 (27/372)	7.4 (55/745)	8.9 (58/654)	7.1 (17/239)	0.676
Premature rupture (%)	3.0 (11/372)	4.2 (31/745)	4.4 (29/654)	4.6 (11/239)	0.662
Placenta previa (%)	2.4 (9/372)	0.9 (7/745)	2.1 (14/654)	1.7 (4/239)	0.211

Data are presented as median (P25, P75) for continuous variable and %(n/N) for categorical variable.

To adjust for the influence of confounding factors, we conducted multiple logistic regression analysis. The included factors were maternal age (continuous variable), paternal age (continuous variable), BMI (continuous variable), gravidity (continuous variable), duration of infertility (continuous variable), type of infertility (primary/secondary infertility), infertility diagnosis (tubal/male/both/others), PCOS (yes/no), basal antral follicle count, basal serum FSH level, fertilization method (IVF/ICSI), endometrial thickness on the first day of progesterone administration (continuous variable) and type of transferred embryos (cleavage embryo/blastocyst). After adjustments for confounding factors, taking the ‘≤12 days group’ as the reference group, the duration of estrogen treatment did not affect the rate of GDM and other neonatal and perinatal outcomes. The specific AOR values with their 95% CIs are presented in **Table 3**.

**Table 3:** Multiple logistic regression analysis to account for confounding variables for neonatal and perinatal outcomes.

	Duration of estrogen treatment before progesterone administration (days)			
	≤12 (Reference)	13-15	16-19	≥20
Preterm birth				
% (n/N)	6.7 (25/372)	7.5 (56/745)	9.2 (60/654)	10.0 (24/239)
AOR (95% CI)	Ref	1.13 (0.69-1.86)	1.40 (0.84-2.33)	1.52 (0.82-2.86)
Low birth weight				
% (n/N)	5.6 (21/372)	4.6 (34/745)	6.3 (41/654)	5.0 (12/239)
AOR (95% CI)	Ref	0.75 (0.42-1.33)	1.01 (0.57-1.79)	0.71 (0.33-1.56)
Small-for-gestational age				
% (n/N)	4.3 (16/372)	4.4 (33/745)	5.2 (34/654)	2.5 (6/239)
AOR (95% CI)	Ref	0.96 (0.51-1.79)	1.05 (0.56-2.00)	0.47 (0.17-1.29)
Macrosomia				
% (n/N)	13.4 (50/372)	16.0 (119/745)	15.1 (99/654)	13.0 (31/239)
AOR (95% CI)	Ref	1.29 (0.90-1.86)	1.27 (0.86-1.86)	1.05 (0.63-1.75)
Large-for-gestational age				
% (n/N)	28.0 (104/372)	26.6 (198/745)	25.4 (166/654)	21.8 (52/239)
AOR (95% CI)	Ref	1.01 (0.75-1.34)	1.01 (0.74-1.36)	0.84 (0.56-1.27)
Gestational hypertension				
% (n/N)	7.3 (27/372)	7.4 (55/745)	8.9 (58/654)	7.1 (17/239)
AOR (95% CI)	Ref	0.96 (0.59-1.57)	1.09 (0.66-1.79)	0.75 (0.38-1.48)
Gestational diabetes mellitus				
% (n/N)	4.0 (15/372)	6.7 (50/745)	6.4 (42/654)	11.3 (27/239)
AOR (95% CI)	Ref	1.48 (0.77-2.82)	1.87 (0.95-3.66)	1.43 (0.64-3.16)
Premature rupture				
% (n/N)	3.0 (11/372)	4.2 (31/745)	4.4 (29/654)	4.6 (11/239)
AOR (95% CI)	Ref	1.46 (0.72-2.97)	1.65 (0.79-3.42)	1.81 (0.74-4.45)
Placenta previa				
% (n/N)	2.4 (9/372)	0.9 (7/745)	2.1 (14/654)	1.7 (4/239)
AOR (95% CI)	Ref	0.49 (0.18-1.33)	0.64 (0.26-1.57)	0.39 (0.10-1.46)

AOR, adjusted odds ratio; CI, confidence interval. Multiple logistic regression model included maternal age, paternal age, BMI, gravidity, duration of infertility, type of infertility (primary/secondary infertility), infertility diagnosis(tubal/male/both/others), PCOS(yes/no), basal antral follicle count, basal serum FSH level, fertilization method (IVF/ICSI), endometrial thickness on the first day of progesterone administration, type of transferred embryos (cleavage embryo/blastocyst) and duration of estrogen treatment.

## Discussion

The purpose of this study was to analyze whether the duration of estrogen treatment before progesterone application affects neonatal and perinatal outcomes of singleton live births after FET via artificial cycles. Through our single-center retrospective cohort study, our results show that the duration of estrogen treatment does not affect offspring and perinatal outcomes.

Globally, the incidence of infertility is as high as 10%, and IVF is an important method of infertility treatment (25, 26). However, adverse pregnancy and perinatal outcomes, such as SGA, LBW, preterm birth, gestational hypertension and gestational diabetes, were increased in IVF/ICSI cycles, even for singleton births (27, 28). However, the exact biological mechanism leading to adverse perinatal outcomes is unclear. Some studies have shown that infertility disease itself is the main reason for the poor perinatal outcome of assisted reproductive technology (ART) singleton offspring (29). However, an increasing number of studies have shown that the process of ART, such as exposure to superphysiological doses of estrogen, may have an adverse effect on perinatal outcomes (20, 30, 31). The mechanism may be that superphysiological doses of estrogen may affect the development of the endometrium rather than the development and quality of the embryo (32, 33). In the artificial cycle of FET, exogenous estrogen is supplemented to cause endometrial hyperplasia while inhibiting the development of dominant follicles. When the endometrium is suitable, exogenous progesterone is supplemented to promote the synchronous development of the endometrium and the embryo. It is not clear whether prolonging the use of estrogen would affect offspring and perinatal outcomes.

To the best of our knowledge, only two studies have analyzed the relationship between the duration of estrogen treatment and neonatal and perinatal outcomes. Bourdon M et al. (13) reported that the mean birth weight and Z scores were significantly lower for the ‘36–48 days’ group than for the ‘≤21 days’ group (3042 ± 801.2 g and −0.44 ± 1.50 vs. 3362 ± 602.9 g and 0.10 ± 0.94, respectively). Another study included only single, vitrified-warmed, euploid blastocysts, and the results showed that variation in the duration of estradiol supplementation before progesterone initiation did not impact frozen, euploid blastocyst transfer outcomes. The duration of estrogen administration was inversely correlated with gestational age at delivery, but this did not translate into an increase in preterm delivery (15). To date, our research is the largest retrospective cohort study to analyze the relationship between the duration of estrogen treatment before progesterone application and neonatal outcomes. This is the first study to analyze the relationship of this duration to pregnancy complications, including hypertensive states of pregnancy, GDM, placenta previa, placental abruption and premature rupture. Through analysis, different estrogen use times had no significant effect on offspring or perinatal outcomes. However, this study also has certain limitations. First, it suffers from the limitation of a retrospective cohort, although we used strict inclusion and exclusion criteria and multiple logistic regression to correct for the influence of confounding factors. Second, regarding the duration of estrogen use, the time range of hormone use at our center is 10-30 days, most of the treatment durations are 13-18 days (25%-75%), and only 275 cases have been treated for more than 19 days; this estrogen use time is shorter than that in Bourdon M’s (13) study, and his research shows that a duration of estrogen treatment greater than 35 days may have an impact on the outcome of offspring mean birth weight. However, in our center, there are almost no transplantation cycles with an estrogen application time greater than 35 days. According to our current data in our reproductive center, the duration of estrogen therapy does not affect offspring and perinatal outcomes, but it is uncertain whether extending the estrogen application time will affect the outcome. Consequently, further analysis of the safety of the estrogen treatment duration before progesterone application is needed for a well-designed, prospective, and large-scale cohort study.

## Conclusion

In conclusion, the duration of estrogen treatment before progesterone application does not affect neonatal and perinatal outcomes in FET cycles. For patients and clinicians, the transplant time can be flexibly arranged for FET in artificial cycles. However, due to the retrospective cohort study limitation, further randomized controlled studies with large samples are needed.

## Data availability statement

The raw data supporting the conclusions of this article will be made available by the authors, without undue reservation.

## Ethics statement

The studies involving human participants were reviewed and approved by the ethics committee of The Third Affiliated Hospital of Zhengzhou University. Written informed consent for participation was not required for this study in accordance with the national legislation and the institutional requirements.

## Author contributions

JZ and LS designed the study and selected the populations to be included and excluded. JZ and MD were involved in the data extraction and analysis. YG reviewed the data. ZW and SW participated in the revision of the manuscript. JZ and MD were involved in drafting this article. All authors contributed to the article and approved the submitted version.
